# Leprosy among children in an area without primary health care coverage in Caratateua Island, Brazilian Amazon

**DOI:** 10.3389/fmed.2023.1218388

**Published:** 2023-06-22

**Authors:** Izabelle Laissa Viana Costa, Patrícia Fagundes da Costa, Sâmela Miranda da Silva, Angélica Rita Gobbo, Pablo Diego do Carmo Pinto, John Stewart Spencer, Moises Batista da Silva, Claudio Guedes Salgado

**Affiliations:** ^1^Laboratório de Dermato-Imunologia, Universidade Federal do Pará, Marituba, Pará, Brazil; ^2^Laboratório de Genética Humana e Médica, Instituto de Ciência Biológicas, UFPA, Belém, Brazil; ^3^Faculdade de Medicina, Instituto de Ciências Médicas, UFPA, Belém, Pará, Brazil; ^4^Department of Microbiology, Immunology and Pathology, Colorado State University, Fort Collins, CO, United States; ^5^Coordenação de Atenção às Doenças Transmissíveis na Atenção Primária à Saúde, Departamento de Gestão do Cuidado Integral, Secretaria de Atenção Primária à Saúde, Ministério da Saúde, Brasília, Distrito Federal, Brazil

**Keywords:** leprosy, children, RLEP qPCR, anti-PGL-I, active case finding

## Abstract

**Introduction:**

The detection of leprosy in children is an important epidemiological marker of the disease, indicating the community’s early exposure to *Mycobacterium leprae* and active transmission of the infection.

**Methods:**

In order to detect new cases among children by combining clinical evaluation and laboratory tests, we conducted an active case finding among individuals under 15 years old on Caratateua Island, located in the city of Belém, in the Pará state, an endemic region in the Amazon. Dermato-neurological examination, collection of 5 mL of peripheral blood for IgM anti-PGL-I antibody titration, and intradermal scraping for bacilloscopy and amplification of the specific RLEP region by qPCR were performed.

**Results:**

Out of the 56 examined children, 28/56 (50%) new cases were identified. At the time of evaluation, 38/56 (67.8%) children presented one or more clinical alterations. Seropositivity was detected in 7/27 (25.9%) new cases and 5/24 (20.8%) undiagnosed children. DNA amplification of *Mycobacterium leprae* was observed in 23/28 (82.1%) of new cases and in 5/26 (19.2%) of non-cases. Out of the total cases, 11/28 (39.2%) were exclusively diagnosed by clinical evaluation performed during the active case finding. Seventeen new cases (60.8%) were detected considering the clinical alterations found in addition to positive results for qPCR. In this group, 3/17 (17.6%) qPCR-positive children presented significant clinical changes 5.5 months after the first evaluation.

**Discussion:**

Our research detected a number of cases 5.6 times higher compared to the total number of pediatric cases recorded throughout the year 2021 in the municipality of Belém, which shows a critical scenario of underdiagnosing of leprosy among children under 15 years old in the region. We propose the use of qPCR technique to identify new cases among children with oligosymptomatic or early disease in endemic areas, in addition to the training of Primary Health Care professionals and the implementation of the Family Health Strategy coverage in the visited area.

## 1. Introduction

Leprosy is an infectious disease that affects mainly peripheral nerves and skin, but also internal organs and the eyes, caused by the *Mycobacterium leprae* bacillus, discovered 150 years ago by the Norwegian doctor Gerhard Armauer Hansen, and by the *Mycobacterium lepromatosis*, described more recently as the foremost causative agent of leprosy in Mexico (1). Despite being one of the oldest known diseases, leprosy continues to affect thousands of people around the world annually, which is related to chronic and historical problems such as lack of diagnosis, misinformation, and social stigma (2).

It is known that the diagnosis of leprosy in childhood, in particular, is closely linked to active transmission foci of the disease in endemic areas (3). In 2021, approximately 9,502 cases among children were reported worldwide, representing a rate equivalent to 4.5 cases per million children (4). In view of these data, one of the main goals proposed by the Global Leprosy Strategy (2021–2030), devised by the World Health Organization (WHO), is to reduce the rate of pediatric leprosy cases by 90% per million children by 2030 (5). However, such a significant reduction goal could also increase underreporting of the disease and hide the true epidemiological scenario of leprosy in the pediatric population.

In Brazil, leprosy continues to be a significant public health problem, affecting children, adults, and elderly individuals of both sexes. Alongside India and Indonesia, Brazil is one of the countries that detects the most cases of the disease in the world. Preliminary data from the Ministry of Health shows that, in 2022, the country recorded 14,962 new cases, with 645 (4.3%) among children under 15 years of age. Pará, located in the Brazilian Amazon and one of the states with the highest number of cases detected per year, diagnosed 1,135 individuals with the disease, considered a highly endemic state, with 13.4 cases per 100,000 inhabitants (6, 7). Among these records, 58 (5.1%) were observed in children under 15 years of age (7).

Belém, the capital of the state of Pará, recorded 158 new cases of leprosy in 2021, of which 5 (3.1%) were detected in children under 15 years of age (8). The Island of Caratateua, also known as Outeiro Island, is located in the city of Belém and is home to approximately 80,000 inhabitants, serving as the headquarters of one of the eight districts of the municipality: the Outeiro Administrative District. This island is an impressive example of explosive population growth, as in 1970 it had only about 1,000 inhabitants, a number that jumped to 15,000 in 1990 and 35,000 in 2010, according to the demographic censuses carried out in the country. With current estimates, the population is around 80 times larger than it was just over 50 years ago (9). Currently, the island represents an important tourist spot in the city, however, it is still characterized by having a socioeconomically vulnerable population (7).

Among the main strategies for combating the disease advocated by the Ministry of Health and WHO is active case finding, which enables the detection of leprosy and immediate treatment of affected individuals (5, 7, 10). Studies have revealed the importance of case screening among schoolchildren as an important mechanism for interrupting the infection transmission chain (11, 12). However, some challenges hinder the establishment and advancement of these, and other strategies related to the disease, including the diagnosis itself. As the diagnosis is predominantly clinical, detecting the disease requires a specialized and experienced professional who recognizes the signs and symptoms that define leprosy. Identifying these characteristics among children can be even more challenging, even among leprologists, given that in many cases, school-age patients do not present a suggestive and evident disease profile, especially in the initial stage. Because they are pediatric patients, the leprologist may also face difficulties in conducting the clinical evaluation of the peripheral nerves (11).

Laboratory techniques have been developed over the past few decades to improve the diagnosis and monitoring of leprosy. Immunological/serological tools, for example, are based on the detection of specific components such as anti-PGL-I antibodies (phenolic glycolipid-I) or the measurement of IFN-γ by T cells of the immune system (13, 14). Previous studies have demonstrated the importance of anti-PGL-I IgM in the serodiagnosis and pathogenesis of leprosy, including the correlation between seropositivity and an increased probability of developing the disease in hyperendemic regions (15, 16).

Another important technique is Real-Time PCR or qPCR (Quantitative Polymerase Chain Reaction), which allows for the amplification of *M. leprae* genetic material in clinical samples. Previous research has demonstrated the efficiency of this method in detecting the pathogen’s DNA among sick individuals (17–20). These findings have made qPCR a promising tool for the diagnosis of the disease, considering its high sensitivity and specificity. It is also important for the diagnosis of difficult cases, such as paucibacillary patients, individuals with atypical clinical manifestations or primary neural cases (17, 21). The gene regions used as targets for the method include the RLEP (*M. leprae-specific repetitive element*), rpoT, Sod A, and 16 s rRNA genes, with the first mentioned target being the most sensitive in relation to the others according to previous studies (18, 22).

Therefore, the objectives of this study were: (a) to detect leprosy cases among children under 15 years of age on an island located in an endemic area in the Brazilian Amazon, and (b) to use the qPCR technique in association with clinical examination to define new cases among evaluated children.

## 2. Materials and methods

### 2.1. Study area

Pará is a state in the northern region of Brazil that is home to an estimated population of 8.7 million people. It is the second-largest state in the country, with an area of 1,245,870.700 km^2^. Belém, the capital of the state, has approximately 1.5 million inhabitants (23), and includes about 39 islands under its administration. Caratateua Island, which has an area of 3.17 hectares, is located 18.8 km from the center of the capital and is characterized by precarious urbanization and higher population density compared to most of the city’s islands. The active case finding of the present study was carried out in an after-school program for low-income children conducted in a facility that provided extracurricular activities and meals to enrolled children, located in a neighborhood (Brasília) without coverage of the Family Health Strategy, an important model integrated into Primary Health Care in Brazil, which provides assistance to families through multidisciplinary teams and healthcare services.

### 2.2. Population and study design

We conducted an active case finding, whose target population consisted of children between 6 and 14 years old enrolled in an after-school program. During the study period, 82 students were enrolled in the location. The active case finding was carried out by a multidisciplinary team in July 2022, with the target population recruited by the program’s coordinator, with authorization from the responsible parties. To participate in the study, children were required to be under 15 years old, regularly enrolled in the after-school program, and have written consent from their guardians. The participants underwent a dermato-neurological examination by a leprologist and the collection of 5 mL of peripheral blood for the titration of IgM anti-PGL-I antibodies by ELISA (*Enzyme-Linked Immunosorbent Assay*), as well as an intradermal scraping of the auricular lobes for bacilloscopy and amplification of the specific RLEP region by qPCR.

### 2.3. Clinical diagnosis

The clinical diagnosis of leprosy was made by a leprologist, based on the recommendations advocated by the World Health Organization, which correspond to the observation of (a) dermatological lesions (hypopigmented or reddish) with loss of sensation and/or (b) thickened or enlarged peripheral nerve with loss of sensation (with or without weakness of the muscles supplied by that nerve); in addition to this, (c) the presence or absence of alcohol-acid resistant bacilli in an intradermal scraping smear was also taken into consideration (24). The simplified modified dermato-neurological assessment form was used to analyze the integrity of neural function, which includes inspection, palpation/percussion, evaluation of sensitivity function and muscle strength associated with nerves, according to the Ministry of Health guidelines (25).

The diagnosed cases were reported, and socioeconomic data were collected through a standardized electronic questionnaire contained in the Hansys software, a system developed by the team at the Dermato-Immunology Laboratory (UFPA) in partnership with the Federal University of West Pará (UFOPA). Individuals registered as cases were notified and referred for treatment with multidrug therapy (MDT) at the nearest health facility.

### 2.4. Laboratory procedures

Samples of intradermal scrapings from the earlobes were fixed on slides for the bacilloscopy technique and also added to microtubes containing 70% alcohol, which were kept at room temperature for total DNA extraction. The fixed slides were subjected to Ziehl-Neelsen staining adapted for the identification *M. leprae*. The bacterial load and bacillary and morphological indices were calculated according to the guidelines of the Ministry of Health (26). For total DNA extraction, the protocol recommended by the manufacturer (Qiagen DNeasy Blood and Tissue kit, Germantown, MD, United States) was used. Amplification of the RLEP region was performed according to the protocol described in a previous study, using primers LP1 (5′-GTGAGGGTAGTTGTT-3′) and LP2 (5’-GGTGCGAATAGTT-3′) (27).

The collected blood samples were refrigerated at 2° - 4° C, and later centrifuged to obtain the plasma used in the ELISA test. The titration of IgM anti-PGL-I antibodies was performed according to a previously described protocol (28). The cut-off for seropositivity was defined by an optical density (OD) equal to 0.295, based on the mean plus three times the standard deviation of healthy individuals from the same endemic area, according to the protocol described in the cited study. The processing of biological samples was carried out at the Dermato-Immunology Laboratory located in Marituba, Pará.

### 2.5. Statistical analysis

The Mann–Whitney test (U) was performed to compare the anti-PGL-I antibody titers between study groups. The Pearson Chi-Square test and Fisher’s exact test were used to analyze categorical variables. Results were considered significant when *p* < 0.05. Statistical analysis and graphing were performed using GraphPad Prism software version 6.0.

### 2.6. Ethical process

This study was approved by the Institute of Health Sciences Research Ethics Committee from Pará Federal University (CAAE 26765414.0.0000.0018 CEP-ICS/UFPA). The participants and their guardians signed the Informed Consent Form, authorizing the conduct of the activity.

## 3. Results

### 3.1. Active case finding among children under 15 years Old

Fifty-six out of 82 (68%) children enrolled in after-school program were evaluated during the active case finding. Among the participants, 29/56 (51.7%) were male, and 50/56 (89.2%) were brown, a self-declared color of skin or ethnic-racial identification, as required by Brazilian laws. Previous DNA studies show a mixed European-Amerindian-African contribution in the formation of Belém population (29). The evaluated children had a mean age of 8.94 years old (±2.28). During the assessment, 28/56 (50%) new cases were diagnosed, of which 15/28 (53.5%) were female, 26/28 (92.8%) were brown, and had a mean age of 9.25 years old (±2.23). Prior contact with individuals diagnosed with the disease was reported in 4/28 (14.2%) of the cases. The presence of the Bacille Calmette-Guérin (BCG) vaccine scar was recorded in 27/28 (96.5%) of the diagnosed children. There were no statistically significant differences observed in the analysis of the variables sex, skin color, presence of BCG scar, and living with leprosy cases (Table 1).

**Table 1:** Epidemiological characteristics of children diagnosed with leprosy.

Characteristics	n/n (%)	*p value*
Sex		**0.593**
Female	15/28 (53.5)	
Male	13/28 (46.5)	
Age range		
6–8	11/28 (39.2)	
9–11	12/28 (42.8)	
12–14	5/28 (12.0)	
Race/color		**0.669**
Brown	26/28 (92.8)	
Black	1/28 (3.6)	
White	1/28 (3.6)	
BCG scar		**0.101**
Presence	27/28 (96.5)	
Absence	1/28 (3.5)	
Living with leprosy cases		**0.669**
Yes	4/28 (14.2)	
No	24/28 (85.8)	

The bold numbers are the *p* values (all non-significant) verified by fisher exact test.

Regarding the diagnosis, primary neural leprosy (i.e., without dermatological lesions, but with involvement of peripheral nerves) and borderline leprosy characterized the clinical form of 20/28 (71.4%) and 8/28 (28.6%) cases, respectively. Ten (35.8%) of the 28 new cases were detected with grade 1 disability and none were detected with grade 2 (Table 2). Six (75%) out of 8 cases classified as borderline leprosy had grade 1 of physical disability. The clinical alterations identified among the new cases can be seen in Table 3. The radial and superficial fibular nerves were altered in 12/28 (42.8%) of the new cases. In addition, loss of muscle strength was observed in 10/27 (37%) cases, with the ulnar nerve being the main affected nerve (37%). Sensory evaluation identified plantar sensitivity alteration in 14/27 (51.8%) of the cases. In 8/28 (28.6%) of the diagnosed children, hypochromic macules with regions of hypoesthesia and/or anesthesia were observed.

**Table 2:** Clinical characteristics of children diagnosed with leprosy.

Characteristics	n/n (%)
WHO operational classification	
Multibacillary	28/28 (100)
Paucibacillary	0/28 (0)
Clinical form	
Primary neural	20/28 (71.4)
Borderline	8/28 (28.6)
Disability grade	
Grade 0	18/28 (64.2)
Grade 1	10/28 (35.8)
Grade 2	0/28 (0)

**Table 3:** Overview of the clinical alterations presented by children diagnosed with leprosy.

Clinical examination	Nerve/region examined	With alteration
		n/n (%)
	Upper limbs	
Inspection/palpation of nerve	Auricular	4/28 (14.3)
	Radial	12/28 (42.8)
	Ulnar	8/28 (28.6)
	Median	2/28 (7.1)
	Lower limbs	
	Tibial	11/28 (39.3)
	Commom fibular	5/28 (17.9)
	Superficial fibular	12/28 (42.8)
	Upper limbs	
Evaluation of muscle strength^*^	Radial	2/27 (7.4)
	Ulnar	10/27 (37.0)
	Median	4/27 (14.8)
	Lower limbs	
	Fibular (Extension)	2/27 (7.4)
	Fibular (Dorsiflexion)	0/27 (0)
	Upper limbs	
Sensory evaluation^*^	Hands	0/27 (0)
	Lower limbs	
	Feet	14/27 (51.8)

*One individual in the group was not submitted to this evaluation.

### 3.2. Laboratory results

**Figure 1:**
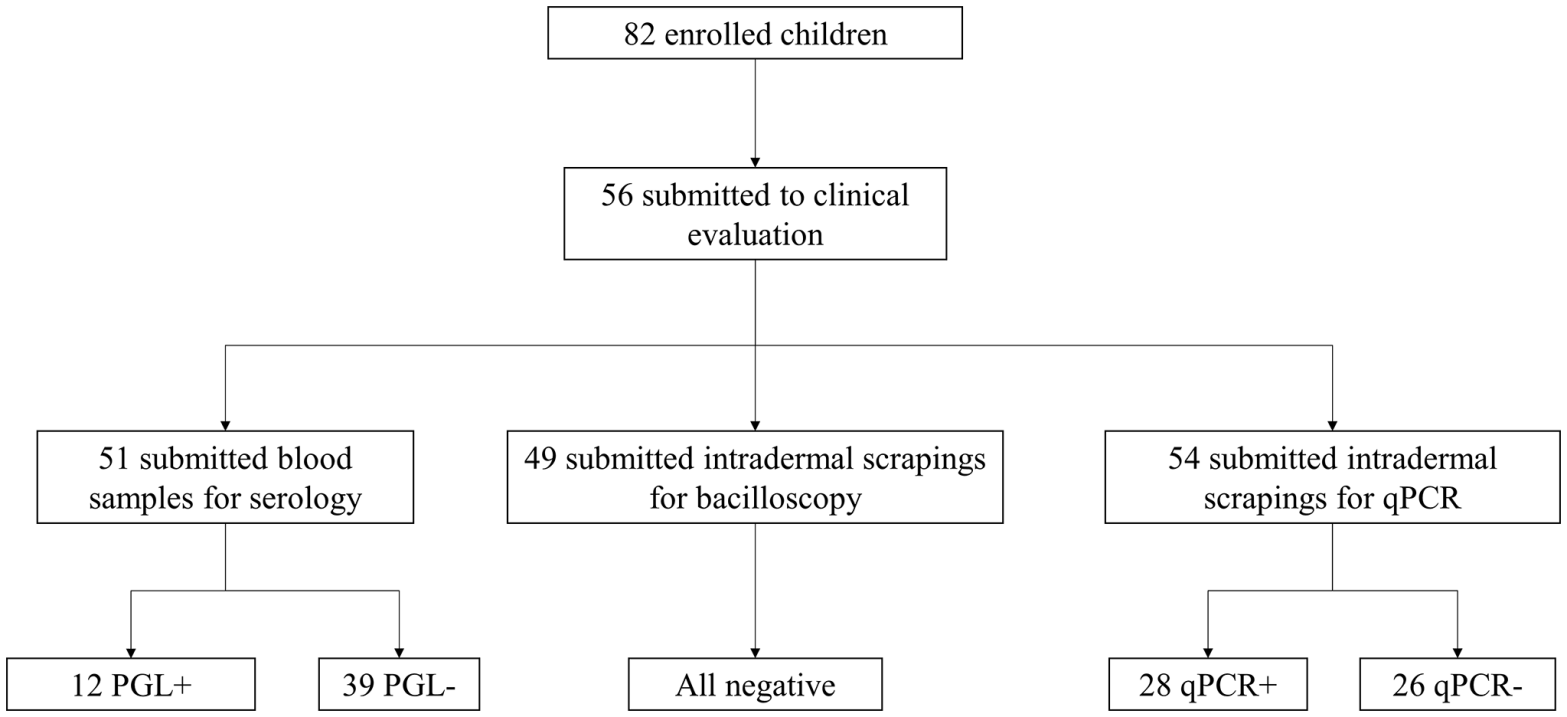
Diagram of conducted laboratory examinations, number of samples collected per test, and overall results.

Figure 1 illustrates the laboratory techniques performed, the quantity of collected samples, and overall results. Considering that some children did not allow blood sample or intradermal scraping collection, the number of samples varied compared to the total number of study participants. Priority was given to qPCR testing over bacilloscopy testing for intradermal scraping samples from individuals for whom collection was challenging.

**Figure 2:**
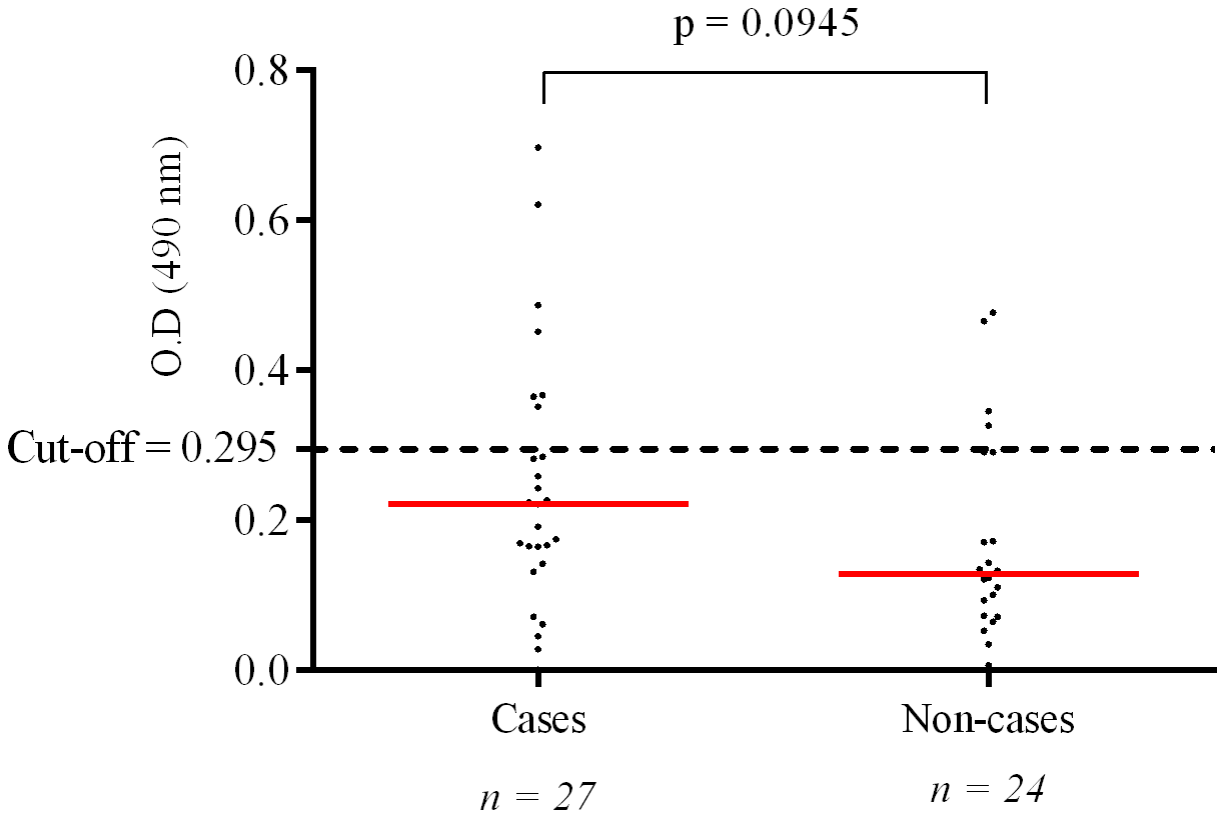
Titration of IgM anti-PGL-I antibodies among children under 15 years old diagnosed and not diagnosed with leprosy. The *p* value was calculated using the Mann–Whitney test for comparison of two unpaired groups with the aid of GraphPad Prism 6 software.

Table 4 shows the correlation between the results obtained for anti-PGL-I IgM titration and qPCR amplification of the specific RLEP region. Of the total number of evaluated individuals who underwent serological testing, 12/51 (23.5%) were seropositive, including 7/27 (25.9%) new cases and 5/24 (20.8%) non-cases. Figure 2 shows the distribution of anti-PGL-I IgM levels between both groups. The mean OD between new cases and non-cases corresponded to 0.222 and 0.128, respectively. There was no statistical difference between the groups.

**Table 4:** Correlation between anti-PGL-I IgM titration and qPCR amplification of the RLEP region.

	Anti-PGL-I IgM		qPCR RLEP		PGL/qPCR
	Positive	Negative	Positive	Negative	Double positivity
	n/n (%)	n/n (%)	n/n (%)	n/n (%)	n/n (%)
New cases	7/27 (25.9)	20/27 (74.1)	23/28 (82.1)	5/28 (17.9)	6/27 (22.2)
Non-cases	5/24 (20.8)	19/24 (79.2)	5/26 (19.2)	21/26 (80.2)	1/24 (4.1)
Total	12/51 (23.5)	39/51 (76.5)	28/54 (51.8)	26/54 (48.2)	7/51 (13.7)

**Figure 3:**
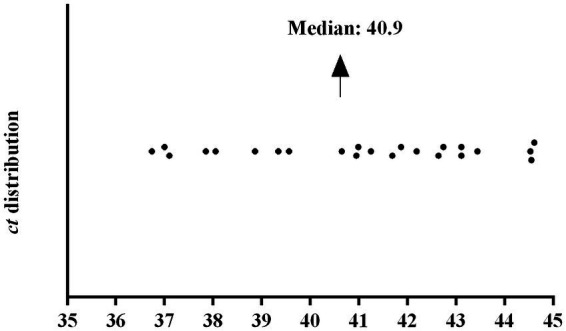
Distribution of *ct* (*cycle threshold*) among 23 qPCR-positive children diagnosed with leprosy in active case finding.

Fifty-four (96.4%) children underwent intradermal scraping collection for qPCR technique and 49/56 (87.5%) also collected material for the bacilloscopy technique. In total, 28/54 (51.8%) individuals tested positive for qPCR, including 23/28 (82.1%) of new cases, which presented an average *ct* (*cycle threshold*) equal to 40.9 cycles (Figure 3). Among non-cases, 5/26 (19.2%) showed positivity for the technique. Double positivity for serological and molecular methods was detected in 6/27 (22.2%) of cases and 1/24 (4.1%) of non-cases. None of the examined children tested positive for the bacilloscopy method.

### 3.3. Combination of clinical and laboratory aspects

**Figure 4:**
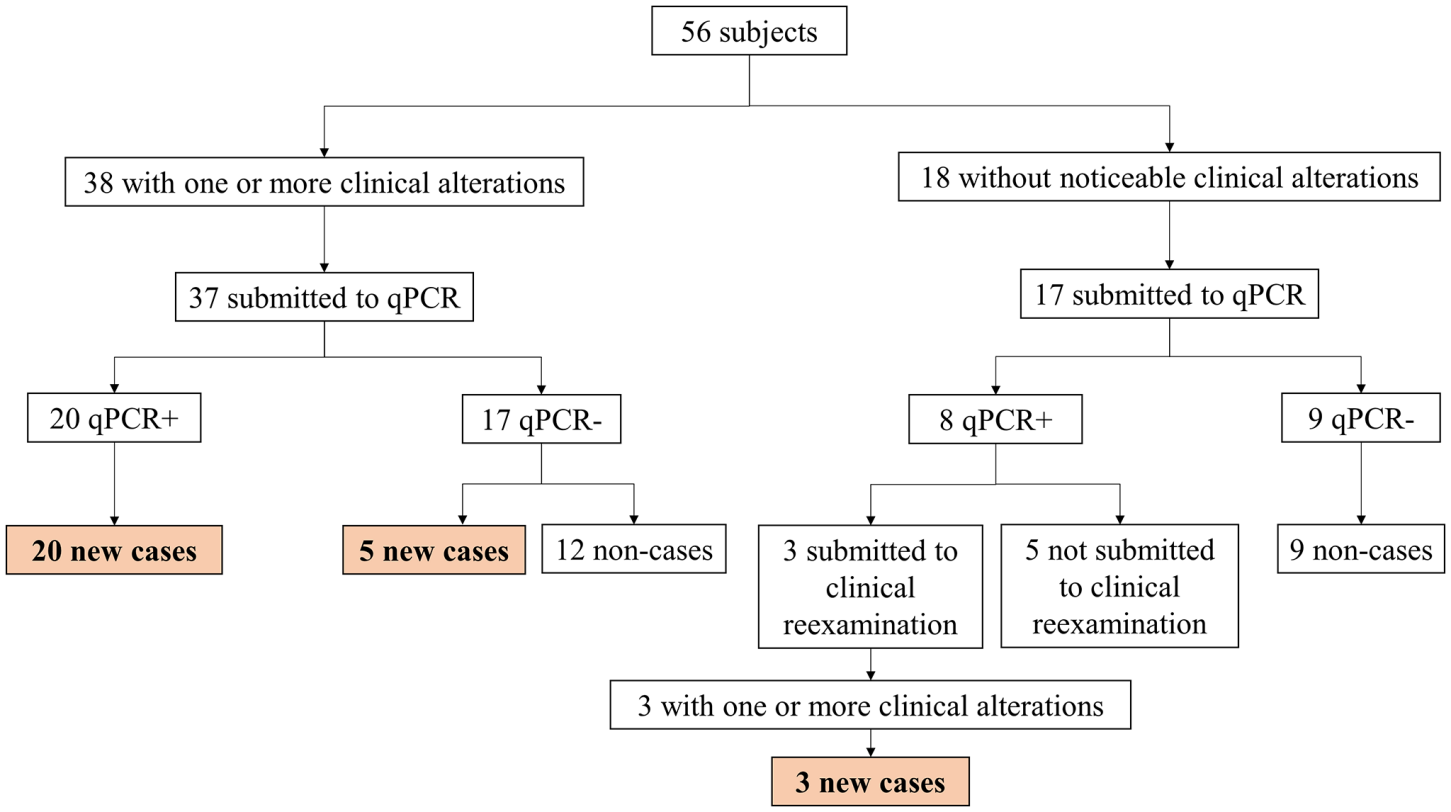
Diagram of the use of qPCR technique as a complementary exam of new cases among children under 15 years old diagnosed in an active case finding.

An overview of the application of the qPCR technique for the diagnosis of new cases can be seen in Figure 4. Among the evaluated children, 38/56 (67.8%) presented at least one clinical alteration during the evaluation. The clinical alterations varied from the unique presentation of pain upon palpation of a peripheral nerve to the loss of muscle strength combined with hypochromic macules and altered sensitivity. Of this group, 20/37 (54%) showed positivity for the qPCR technique. At the time of clinical evaluation by the leprologist, 11/56 (19.6%) children were diagnosed with the disease, of which 6/11 (54.5%) were positive for the qPCR technique. The remaining diagnosed cases, which correspond to 17/28 (60.7%), were defined based on the presence of one or more clinical alterations recorded in the evaluation, added to a positive result in the qPCR technique. Three of these 17 patients (17.6%) did not present clinical alterations during the active case finding carried out in July 2022, and suggestive alterations of leprosy were found approximately 5.5 months after the first dermato-neurological examination, during a reassessment.

## 4. Discussion

Early detection and treatment are essential strategies to break the transmission chain of leprosy, with case finding in the community being the ideal way to achieve them. The present active case finding was carried out after a request from the coordination of an after-school program due to the observation of children with suggestive dermatological lesions and prior knowledge of leprosy cases in families in the region. The Island of Caratateua, like the other inhabited islands in the capital of Pará, is characterized by having a socioeconomically vulnerable population, with the after-school program being a place of shelter and offering extracurricular activities mainly aimed at children from low-income and at-risk families. Historically, the area has experienced intense disorderly occupation by people looking for permanent or holiday housing, which has resulted in substandard housing (9, 30, 31).

During the active search action, 28 out of 56 children under 15 years old were diagnosed with leprosy. This number is 5.6 times higher than the number of pediatric cases registered throughout the year 2021 in Belém, revealing the huge underdiagnosing of the disease in the municipality, which is classified as highly endemic, with 10.49 cases per 100,000 inhabitants (7, 8).

Previous studies by our research group have demonstrated the hidden prevalence of leprosy cases in hyperendemic municipalities in the state of Pará (12, 18, 27, 32, 33). It is believed that the data found in this research may still be underestimated, given that 67.03% of the population of Belém remains without coverage of primary care services, which is responsible for conducting essential actions to fight leprosy, such as active case finding and assistance to people affected by the disease (33–35). The area where the active case finding was conducted, in particular, is not covered by the Family Health Strategy, the priority model of Primary Health Care in the country (36). In addition, the Covid-19 pandemic has further exacerbated the concerning scenario of underdiagnosing of leprosy in Brazil and worldwide, by hindering patients’ access to the health services and consequently negatively impacting the number of new diagnoses (4).

It was observed that 10 out of 28 (35.8%) cases had grade 1 physical disability in this study. One of the main characteristics of leprosy is its long incubation period (between 2 and 5 years), which can reach decades in some cases, resulting in a diagnosis that is predominantly made in adults. This can lead to the mistaken idea that diagnosis in children corresponds to early diagnosis. The presented data show that a significant portion of cases was diagnosed late, considering that the detection of physical disabilities is closely linked to delay in leprosy detection. The WHO Global Leprosy Strategy (2021–2030) proposes as a priority to reduce to zero the number of new pediatric patients with physical disabilities by 2030, which will require effective strategies of epidemiological surveillance aimed at early diagnosis and contact tracing in endemic areas (5).

Late diagnosis among children can be multifactorial, including the inability of health professionals to adequately detect the disease. In addition to this fact, a study conducted in tertiary hospitals in India observed that among the main risk factors associated with delayed diagnosis (represented by physical disability and/or positive bacilloscopy), socioeconomic vulnerability was a factor that increased the possibility of delayed diagnosis by 6 times (37). Socioeconomic aspects are deeply associated with the higher risk of development and progression of leprosy, and these factors can also be attributed to the high number of new cases found and the detection of physical disabilities in more than 30% of children diagnosed during this study (38, 39). In addition to facing the disease, patients diagnosed with leprosy are often subjected to social discrimination, historically linked to the infection (2). Regarding leprosy in childhood, deprivation of education, bullying, and rejection due to stigma can occur (40), especially among children with visible disabilities caused by the disease, however, the lack of studies on this aspect makes it difficult to analyze the real impact of the diagnosis on the social life of pediatric patients (41).

Studies conducted in the Comoros Islands, off the southeast coast of Africa, have revealed the persistent hyperendemicity of leprosy in the population, despite efforts to control the disease over the past 40 years (42, 43). Hasker and colleagues (42) observed that between 2000 and 2015, the trend of increasing numbers of new diagnoses accompanied the period of intensified active case finding activities on the island of Anjouan in the Comoros. Diagnosis among children accounted for an average of 33% of total cases during this period, indicating active transmission of the infection in communities that are marked in part by social inequality and difficulty accessing adequate primary healthcare services (44).

Kiribati, an island nation located in Oceania, achieved the goal of eliminating leprosy as a public health problem in the year 2000 by presenting a prevalence of 0.94 cases per 10,000 inhabitants. However, since then, it has observed a growth in the number of cases above the previously achieved goal, particularly among children, a scenario attributed to increased efforts to detect new cases through active case finding. Of the 2,287 new cases diagnosed in the archipelago between 1988 and 2017, 757 (33%) were registered in individuals under 15 years of age (45). A previous study conducted by our research group on one of the islands in the city of Belém (Mosqueiro Island) identified 65 new cases among 706 (9.6%) schoolchildren evaluated, which evidenced the hidden prevalence of leprosy cases in the area. Like Caratateua Island, Mosqueiro Island also has low coverage of Family Health Strategy services (27).

In addition to clinical aspects, the diagnosis of leprosy can be aided by laboratory tests. Among these, bacilloscopy is considered the gold standard. However, despite its high specificity, the method has low sensitivity, especially in early cases, in paucibacillary cases and in cases of primary neural leprosy (18), which was the predominant form of cases in this study (71.4%). Retrospective studies on the diagnosis of pediatric cases observed positivity in the bacilloscopy method above 50% in Cuba (46) and 80% in Nepal (47), suggesting a concerning dependence on this technique for the diagnosis and detection of cases in more advanced stages in these countries, which have already declared the elimination of leprosy as a public health problem.

As a strategy to improve the ability to detect cases early, laboratory tools such as serological and molecular biology tests have been employed as important biomarkers for the disease. Previous studies have shown an important association between seropositivity for anti-PGL-I antibody titers and a higher risk of developing leprosy in the future in hyperendemic areas of Pará (15). In the present study, 7/27 (25.9%) of the cases tested positive for the serological test. The observed seroprevalence was similar to that found among non-cases (20.8%), which may be attributed to the clinical forms presented by the patients (borderline and primary neural leprosy), which are further from the lepromatous pole, known to be related to high levels of anti-PGL-I IgM antibodies due to the predominance of the humoral immune response.

In addition to the serological method, amplification of the repetitive RLEP region by qPCR showed high ability to detect *M. leprae* genetic material in leprosy patients in previous studies (11, 17, 19, 20), which led to the proposal of submitting individuals who are doubly positive for serological and molecular techniques to treatment for the disease, considering their situation of subclinical infection and potential for maintaining bacillary proliferation in the community (18). Among the new cases in this study, 23/28 (82.1%) tested positive for the qPCR technique. This study emphasizes the use of qPCR as an important biomarker for the diagnosis of leprosy in children in an endemic region, especially in the presence of oligosymptomatic or early disease. Most of the diagnosed cases did not present dermatological lesions, with peripheral nerve changes predominating, highlighting the primary neural character of leprosy.

The positivity in the qPCR technique was also observed among 8/17 (47%) children who initially showed no noticeable clinical alterations in the dermatoneurological examination. The research team proposed the re-evaluation of these individuals. About 5.5 months after the first evaluation, 3/8 (37.5%) of the children attended to be reassessed by the leprologist, who detected important clinical alterations, including pain and tingling when palpating peripheral nerves, loss of hand muscle strength, and hypochromic macules with regions of anesthesia. Given the clinical picture, together with the positivity of qPCR, the children were classified as new cases. The positivity in the molecular biology technique prior to the appearance of noticeable clinical manifestations suggest early detection of leprosy. We intend to carry out the reassessment of the five children who had no previous clinical alterations but tested positive in the qPCR technique (who did not attend the initially proposed reassessment) as soon as possible, in order to investigate whether they have become new cases or not.

Our study demonstrated a high number of hidden cases among schoolchildren on an island located in Belém, capital of Pará state, Amazon Region, where leprosy is endemic. We propose the use of qPCR technique for the definition of new cases based on the association between clinical alterations and positivity for the method among children under 15 years old in endemic areas. In addition, we emphasize the need for training of health professionals for the detection of leprosy and the vitalness of increasing Primary Care coverage in the municipality, which will allow for the enhancement of efforts made for the diagnosis of leprosy in childhood, breaking the chain of disease transmission, and preventing affected children from progressing to physical disabilities.

## Data availability statement

The raw data supporting the conclusions of this article will be made available by the authors, without undue reservation.

## Ethics statement

The studies involving human participants were reviewed and approved by Institute of Health Sciences Research Ethics Committee from Pará Federal University (CAAE 26765414.0.0000.0018 CEP-ICS/UFPA). Written informed consent to participate in this study was provided by the participants’ legal guardian/next of kin.

## Author contributions

The study concept was designed by IC, PC, and CS. Clinical examination of sensory-motor functions was performed by SS, PC, and CS. The datasheet with all clinical and epidemiological information was filled and managed by IC. Serological experiments were performed and analyzed by PC, AG, and JS. Antigens used in serological experiments were generously provided by JS. Molecular experiments were designed, managed, and performed by PC and MS. The manuscript was primarily written by IC and PC with substantial critical revision by CS. All authors contributed to the article and approved the submitted version.

## Funding

This work was supported by CNPq (428964/2016-8 grant and 313633/2018-5 fellowship for CS), CAPES PROAMAZONIA 3288/2013, Brazil Ministry of Health 035527/2017, PROPESP/UFPA, VALE S.A. 27756/2019, Fulbright Scholar to Brazil (JS), and the Heiser Program of the New York Community Trust for Research in Leprosy (Josafá Barreto, MS, CS, and JS) grants P15-000827, P16-000796, and P18-000250. The funders had no role in study design, data collection, analysis, interpretation, or writing of the report.

## Conflict of interest

The authors declare that the research was conducted in the absence of any commercial or financial relationships that could be construed as a potential conflict of interest.

## Publisher’s note

All claims expressed in this article are solely those of the authors and do not necessarily represent those of their affiliated organizations, or those of the publisher, the editors and the reviewers. Any product that may be evaluated in this article, or claim that may be made by its manufacturer, is not guaranteed or endorsed by the publisher.
